# Treatment of Recurrent Posttransplant Lymphoproliferative Disorder with Autologous Blood Stem Cell Transplant

**DOI:** 10.1155/2015/801082

**Published:** 2015-11-25

**Authors:** Bharat Malhotra, Ahmad K. Rahal, Hussam Farhoud, Dennis F. Moore, K. James Kallail

**Affiliations:** ^1^Department of Internal Medicine, University of Kansas School of Medicine-Wichita, 1010 N. Kansas Street, Wichita, KS 67214, USA; ^2^Heartland Cardiology, Heartland West, 9000 W. Central, Wichita, KS 67212, USA; ^3^Cancer Center of Kansas, 818 N. Emporia, No. 403, Wichita, KS 67214, USA

## Abstract

*Background*. Posttransplant lymphoproliferative disorders (PTLDs) occur after solid organ transplantation. Treatment guidelines include reduction in immunosuppression (RIS), radiation, rituximab, chemotherapy, and immunological agents. We present a rare case of recurrent diffuse large B-cell lymphoma presenting as a PTLD in a heart transplant patient treated with autologous blood stem cell transplant (ASCT) after failure of conventional therapy.* Case Presentation*. A 66-year-old male presented with a neck mass. He has a history of Hodgkin's disease status after staging laparotomy with splenectomy and heart transplantation due to dilated nonischemic cardiomyopathy 8 years prior to the development of PTLD. His examination was remarkable for right submandibular swelling. An excisional biopsy confirmed the diagnosis of diffuse large B-cell NHL. Patient received RIS, rituximab, chemotherapy, and radiation therapy with a complete remission. His lymphoma relapsed and he subsequently was treated with RICE salvage chemotherapy and consolidative high-dose chemotherapy with BEAC regimen followed by ASCT resulting in a complete remission.* Conclusion*. Patients with PTLD present a difficult therapeutic challenge. In this case, the patient's prior history of Hodgkin's disease, splenectomy, and a heart transplant appear to be unique features, the significance of which is unclear. ASCT might be a promising therapy for patients with relapsed or refractory PTLD.

## 1. Introduction

Posttransplant lymphoproliferative disorders (PTLDs) are a heterogeneous group of benign and malignant entities that occur in the setting of solid organ or allogeneic hematopoietic cell transplantation in the setting of immunosuppression [1]. PTLD is the most common cause of cancer-related mortality in patients with solid organ transplantation [2]. About 85% of PTLDs in the United States are of B-cell origin. The optimal treatment of PTLD is not clearly defined due to a lack of randomized phase III trials. Current management options include reduction in immunosuppression (RIS), surgery, radiation, rituximab, chemotherapy, and antiviral and immunological agents. We present an unusual case of diffuse large B-cell type Non-Hodgkin's Lymphoma (NHL) presenting as a PTLD in an adult with a history of Hodgkin's disease, splenectomy, and heart transplant treated with autologous blood stem cell transplant (ASCT) after failure of conventional therapy.

## 2. Case Presentation

**Figure 1:**
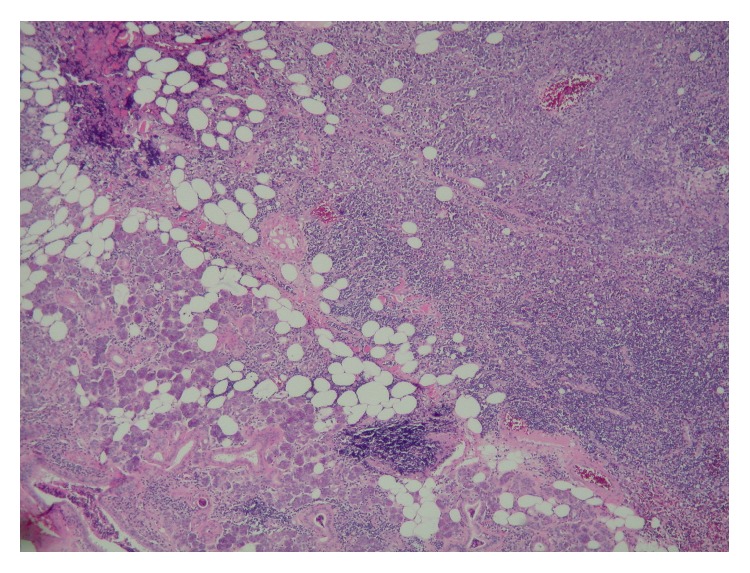
Hematoxylin and eosin stain showing salivary gland and lymphoid components. Small lymphocytes on the left and large lymphocytes on the right.

**Figure 2:**
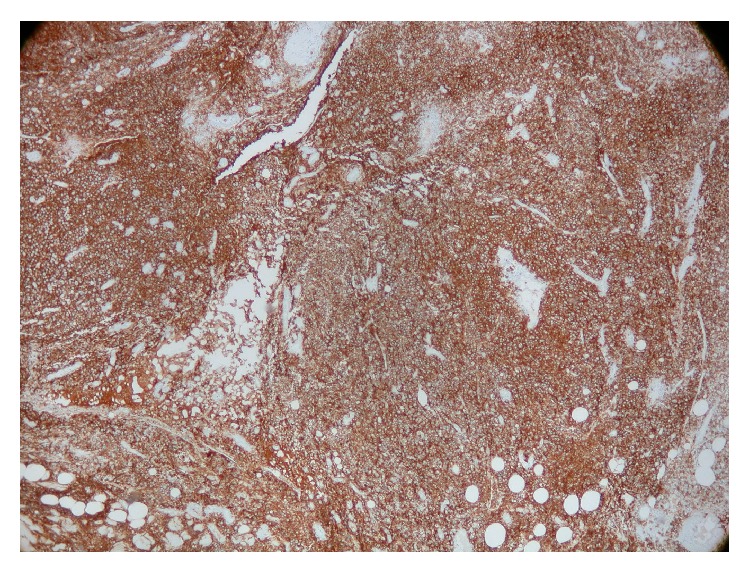
CD 20 immunostain showing 80–90% large lymphoid cells.

**Figure 3:**
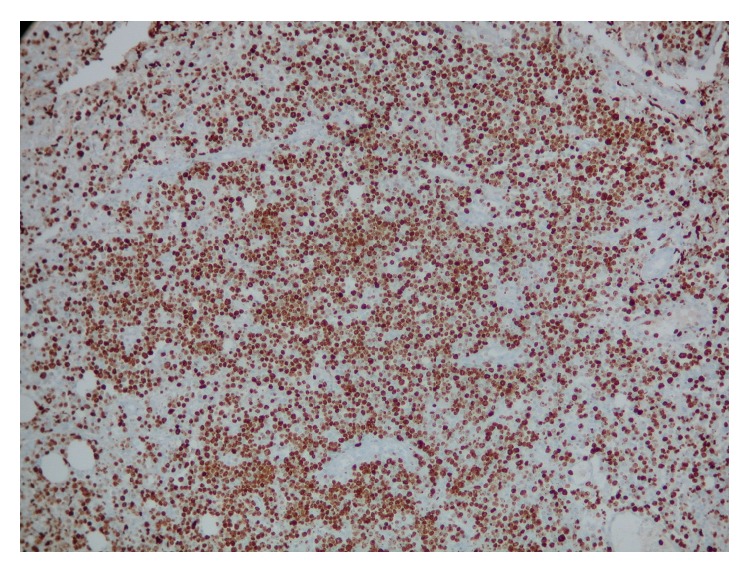
Ki-67 immunostain showing high proliferation rate.

A 66-year-old male patient with a past medical history of dilated nonischemic cardiomyopathy status after heart transplant, immune thrombocytopenic purpura, hypertension, and dyslipidemia presented with a gradually enlarging right neck mass. On physical examination, there was a swelling in the right preauricular and submandibular lymph nodes extending into the cervical nodal area. Laboratory data showed mild leukocytosis but were otherwise unremarkable. His immunosuppressive medications included prednisone 10 mg once daily, tacrolimus 1.5 mg twice a day, and mycophenolate 750 mg twice a day. His workup led to a fine needle aspiration biopsy which was nondiagnostic. An excisional lymph node biopsy confirmed the diagnosis of diffuse large B-cell type NHL germinal center cell subtype (Figure 1). Immunohistochemical studies showed positive staining for CD20 (Figure 2) with high Ki-67 score of approximately 90% (Figure 3). Workup for his NHL included a computerized tomography (CT) scan of neck, chest, abdomen, and pelvis which showed lymphadenopathy in the right parotid and submandibular areas extending anteriorly toward the floor of the mouth, as well as in the right cervical region. Positron Emission Tomography (PET) scan showed corresponding hypermetabolic uptake in the neck. Bone marrow biopsy and aspiration were negative. Human immunodeficiency virus, Epstein-Barr virus (EBV), cytomegalovirus, and hepatitis panel were negative. The stage of his primary lymphoma was Ann Arbor Stage IA [3].

Per recommended guidelines for PTLD treatment [2], RIS was initiated. Prednisone was reduced from 10 mg to 5 mg daily, tacrolimus was reduced from 1.5 mg to 0.5 mg twice a day, and mycophenolate was stopped. There were no further changes in the immunosuppressive treatment during the investigational time, nor was there a change from tacrolimus to m-Tor inhibitors. The patient received six cycles of chemotherapy with rituximab, cyclophosphamide, doxorubicin, vincristine, and prednisone (R + CHOP). Physical examination, complete blood count (CBC), complete metabolic panel (CMP), lactate dehydrogenase (LDH), CT scans, and PET-CT scans confirmed the patient to be in complete remission. Remission duration was brief, four weeks later with biopsy proving local relapse occurring. He received salvage chemotherapy with rituximab, ifosfamide, carboplatin, and etoposide (RICE) chemotherapy. Unfortunately, after 2 cycles of RICE the disease progressed and the mass had become ulcerated and necrotic. Repeat PET scan showed local relapse and he was treated with radiation therapy at standard dose plus fractionation. Despite an initial response, the mass progressed again during radiation therapy. Subsequent treatment with rituximab, gemcitabine, and oxaliplatin (R + GEMOX) resulted in a partial remission. With demonstrated chemosensitivity of his primary refractory disease, he proceeded with high-dose chemotherapy regimen of carmustine, etoposide, cytarabine, and cyclophosphamide (BEAC) with ASCT. Following the ASCT, the patient achieved a complete clinical remission and experienced no recurrence of the PTLD NHL.

## 3. Discussion

Posttransplantation lymphomas were first described in 1968 [4]. Patients with PTLD present a difficult clinical challenge. PTLD may develop at any time following organ transplantation; however, its risk is highest during the first year [1]. Incidence of PTLD after heart transplantation is around 1–6% [5]. The risk for PTLD can be reduced by limiting patient exposure to aggressive immunosuppressive regimens [1, 2]. The pathogenesis of PTLD in most patients appears to be related to B-cell proliferation induced by infection with EBV in the setting of chronic immunosuppression and decreased T-cell immune surveillance [6]. It may be worth considering the additional impact on immune surveillance of a prior diagnosis of Hodgkin's lymphoma and splenectomy, as in this patient. The prognosis is favorable in early PTLD and poor in late PTLD.

The treatment of PTLD is a complex task requiring special considerations. The hematooncology subgroup of the British Committee for Standards in Hematology and the British Transplantation Society recently has made recommendations for the management of PTLD in adult recipients of solid organ transplants, based on literature data and the experience of PTLD specialists [2]. They did not include ASCT in their recommendations for treating PTLD. Treatment options of PTLD are RIS, rituximab, rituximab plus chemotherapy, surgery, radiotherapy, adoptive T-cell therapy, and antiviral and immunological agents [2]. Despite these treatments, the overall mortality of PTLD after solid organ transplantation is around 50% [7]. The rationale of using ASCT in our patient was due to failure of conventional chemotherapy and radiation therapy. The evidence for high-dose chemotherapy and stem cell transplant comes from the PARMA study [8]. Philip et al. [9] showed that, compared with conventional chemotherapy, treatment with high-dose chemotherapy and autologous bone marrow transplantation increases event-free and overall survival in patients with chemosensitive Non-Hodgkin's lymphoma in relapse.

With respect to the cardiac performance at remission and at follow-up, there was no evidence of allograft dysfunction determined by surveillance echocardiogram and cardiac catheterization with an ejection fraction of more than 55%. Clinically, patient overall status after ASCT and on follow-up remained stable at New York Heart Association (NYHA) Functional Classification Classes I-II [10]. The patient did not have any signs of severe allograft rejection at any point and this was verified status after right ventricle endomyocardial biopsy. The worse rejection the patient had according to the International Society of Heart and Lung Transplantation (ISHLT) was Grade 1R [11–13]. Grade 1R is generally not treated unless there is concomitant hemodynamic dysfunction [14, 15]. Our patient was always hemodynamically stable and was never treated for mild allograft rejection (Grade 1R). The follow-up time after ASCT was 17 months.

## 4. Conclusion

PTLD is a serious complication of solid organ transplantation, contributing significantly to morbidity and mortality in this patient group. To our knowledge, treatment with ASCT for PTLD following heart transplant has not been reported before. ASCT might be a promising therapy for PTLD after solid organ transplant that is resistant to recommended therapy, and data from future randomized clinical trials will be important.
